# 26.2 CORTICAL STRESS REGULATION IS DISRUPTED IN SCHIZOPHRENIA BUT NOT IN CLINICAL HIGH-RISK FOR PSYCHOSIS

**DOI:** 10.1093/schbul/sby014.107

**Published:** 2018-04-01

**Authors:** Romina Mizrahi, Alan Wilson, Sylvain Houle, Christin Schifani

**Affiliations:** 1 University of Toronto & Centre for Addiction and Mental Health; 2 PET Centre, Centre for Addiction and Mental Health; 3 University of Toronto

## Abstract

**Background:**

While striatal dopamine in psychosis and stress has been well studied, the role of dopamine in the prefrontal cortex (PFC) is poorly understood. To date no study has investigated the PFC dopamine response to stress exclusively in schizophrenia or its putative prodrome, even though medial PFC is known as a key area in stress regulation. The present study uses the high-affinity dopamine D2/3 receptor radiotracer [11C]FLB457 and positron emission tomography (PET) together with a validated psychosocial stress challenge to investigate if the PFC dopamine response to stress is dysregulated in schizophrenia and clinical high risk (CHR) for psychosis.

**Methods:**

Fourteen antipsychotic-free patients with schizophrenia, 14 CHR and 12 matched healthy volunteers underwent two [11C]FLB457 PET scans, one while performing a Sensory Motor Control Task (control) and another while performing the Montreal Imaging Stress Task (stress). PET data were analyzed using the Simplified Reference Tissue Model with non-displaceable binding potential (BPND) as outcome measure. Dopamine release was defined as percent change in BPND between control and stress scan (ΔBPND).

**Results:**

We observed an increased dopamine release, indexed by ΔBPND, in the medial PFC in schizophrenia patients but not CHR compared to healthy volunteers. Further, associations between stress-induced dopamine release and increase in cortisol levels observed in healthy volunteers and CHR, were absent in schizophrenia, similar to associations with symptoms, distress and anxiety.

**Discussion:**

These findings provide first direct evidence of a disrupted cortical dopamine-stress regulation in schizophrenia.

